# Supplementary material for: Perceptions of Australia’s e-cigarette regulations and recommendations for future reforms: a qualitative study of adolescents and adults
Source: BMJ Open. 2024 Feb 5;14(2):e081032. doi: 10.1136/bmjopen-2023-081032 (PMC10860001; doi:10.1136/bmjopen-2023-081032)
Supplement: Supplementary data [file bmjopen-2023-081032supp001.pdf]

Supplementary Material

Table S1. Coding Hierarchy

Parent code	Child code #1	Child code #2	Child code #3
Perspectives on current regulations	Perspectives on the prescription model	Positive sentiment	Should reduce use among young people and those who have never smoked
		Negative sentiment	E-cigarettes are not a viable quitting aid
			Barriers to access vs. the system is easy to manipulate
			Illusion of product safety
	Perspectives on the availability of non-nicotine e-cigarette products	Positive sentiment	Libertarian views – adults should be allowed to choose what they consume
			Non-nicotine products as potential quitting aids
		Negative sentiment	Safety and addiction concerns
			Nicotine content concerns
			Gateway to nicotine use
			Difficult to enforce differential regulations for nicotine and non-nicotine products
Recommendations for potential regulations	Demand factors	Product and packaging	Health warnings on product packaging

			Plain packaging
			Remove flavours
			Provide detailed information about the contents of e-cigarettes
		Increase price of e-cigarettes	-
		Increase the number of vape-free areas	-
		Limit advertising	Reduce visibility
			Stop appealing to children/young people
	Supply factors	Ban e-cigarettes	Ban importation of all e-cigarettes
			Ban disposable e-cigarettes
		Make e-cigarettes less available to children	-
		Legalise access to e-cigarettes for adults	-
	Miscellaneous	Increase support for mental health and addiction	-
		Enforce regulations	-
