# Supplementary material for: Perceptions of Australia’s e-cigarette regulations and recommendations for future reforms: a qualitative study of adolescents and adults
Source: BMJ Open. 2024 Feb 5;14(2):e081032. doi: 10.1136/bmjopen-2023-081032 (PMC10860001; doi:10.1136/bmjopen-2023-081032)
Supplement: Author's manuscript [file bmjopen-2023-081032.draft_revisions.pdf]

BMJ Open is committed to open peer review. As part of this commitment we make the peer review history of every article we publish publicly available.

When an article is published we post the peer reviewers' comments and the authors' responses online. We also post the versions of the paper that were used during peer review. These are the versions that the peer review comments apply to.

The versions of the paper that follow are the versions that were submitted during the peer review process. They are not the versions of record or the final published versions. They should not be cited or distributed as the published version of this manuscript.

BMJ Open is an open access journal and the full, final, typeset and author-corrected version of record of the manuscript is available on our site with no access controls, subscription charges or pay-per-view fees (http://bmjopen.bmj.com).

If you have any questions on BMJ Open's open peer review process please email info.bmjopen@bmj.com

BMJ Open

Perceptions of Australia's e-cigarette regulations and recommendations for future reforms: A qualitative study of adolescents and adults

Journal:	*BMJ Open*
Manuscript ID	bmjopen-2023-081032
Article Type:	Original research
Date Submitted by the Author:	17-Oct-2023

Complete List of Authors:	Brierley, Mary-Ellen; The University of Melbourne, School of Psychological Sciences<br>Yaw, Sean; The University of Melbourne; The University of Melbourne<br>Jongenelis, Michelle I. ; University of Melbourne VCCC

Keywords:	PUBLIC HEALTH, Health policy < HEALTH SERVICES ADMINISTRATION & MANAGEMENT, Adolescents < Adolescent, QUALITATIVE RESEARCH

SCHOLARONE™
Manuscripts

I, the Submitting Author has the right to grant and does grant on behalf of all authors of the Work (as defined in the below author licence), an exclusive licence and/or a non-exclusive licence for contributions from authors who are: i) UK Crown employees; ii) where BMJ has agreed a CC-BY licence shall apply, and/or iii) in accordance with the terms applicable for US Federal Government officers or employees acting as part of their official duties; on a worldwide, perpetual, irrevocable, royalty-free basis to BMJ Publishing Group Ltd ("BMJ") its licensees and where the relevant Journal is co-owned by BMJ to the co-owners of the Journal, to publish the Work in this journal and any other BMJ products and to exploit all rights, as set out in our licence.

The Submitting Author accepts and understands that any supply made under these terms is made by BMJ to the Submitting Author unless you are acting as an employee on behalf of your employer or a postgraduate student of an affiliated institution which is paying any applicable article publishing charge ("APC") for Open Access articles. Where the Submitting Author wishes to make the Work available on an Open Access basis (and intends to pay the relevant APC), the terms of reuse of such Open Access shall be governed by a Creative Commons licence – details of these licences and which Creative Commons licence will apply to this Work are set out in our licence referred to above.

Other than as permitted in any relevant BMJ Author's Self Archiving Policies, I confirm this Work has not been accepted for publication elsewhere, is not being considered for publication elsewhere and does not duplicate material already published. I confirm all authors consent to publication of this Work and authorise the granting of this licence.

For peer review only

Perceptions of Australia's e-cigarette regulations and recommendations for future reforms: A qualitative study of adolescents and adults

Mary-Ellen E. Brierley[a] , Sean J. L. Yaw[a,b], and Michelle I. Jongenelis[a*]

[a]Melbourne Centre for Behaviour Change, Melbourne School of Psychological Sciences,

The University of Melbourne,

Grattan Street, Parkville, 3010

Australia

[b]Melbourne School of Population and Global Health, The University of Melbourne,

Grattan Street, Parkville, 3010

Australia

*CORRESPONDING AUTHOR

Michelle.jongenelis@unimelb.edu.au

+61 03 9035 4979

ORCID: 0000-0002-0717-1692

Word count

Abstract: 251

Main manuscript: 4342

For peer review only

For peer review only - http://bmjopen.bmj.com/site/about/guidelines.xhtml

Perceptions of Australia's e-cigarette regulations and recommendations for future reforms: A qualitative study of adolescents and adults

Abstract

Objective: To assess public perceptions of the effectiveness of e-cigarette regulations in minimising use among adolescents and those who have never smoked. Specifically, we explored (i) perceived effectiveness of current regulations relating to e-cigarettes and (ii) ideas for further regulations that could reduce use.

Design and participants: Focus groups (*n* = 16) were conducted with Australian adolescents (14 to 17 year olds), young adults (18 to 24 year olds), and adults (25 to 39 year olds). Groups were stratified by age, gender, and e-cigarette use status. Data were analysed using reflexive thematic analysis.

Setting: Focus groups were conducted in-person in two major Australian cities.

Results: Groups lacked a comprehensive understanding of e-cigarette regulations in Australia. When informed of these regulations, half of the groups considered the prescription model for nicotine e-cigarette products to be effective when enforced appropriately. Almost all groups considered adult access to non-nicotine products problematic. All groups suggested a range of demand reduction regulations, including plain packaging, health warnings, flavour restrictions, and increased vape-free areas. Most groups (predominantly those who had never vaped) also recommended supply reduction regulations such as banning all e-cigarettes. The need for supply reduction measures to include addiction and mental health supports was discussed.

Conclusions: The regulations recommended by participants largely align with those that are to be introduced in Australia, indicating that these reforms are likely to be accepted by the public. Ensuring these reforms are complemented by formal supports for young people experiencing nicotine dependence and related mental health concerns is critical.

Keywords: e-cigarettes; regulations; policy; perceived effectiveness; nicotine; adolescents; young adults; adults.

For peer review only - http://bmjopen.bmj.com/site/about/guidelines.xhtml

Article summary: Strengths and limitations of this study

- This was the first study to explore perceptions of e-cigarette regulation effectiveness, with prior work in this space assessing regulation support only.
- We recruited participants across the community and stratified groups by age, gender, and vaping status to gather a broad range of perspectives and explore differences between groups.
- Due to the emergent nature of the coding process, only one researcher coded the data, which prevented the calculation of inter-coder reliability.
- Findings only represent the perspectives of the 139 participants who attended the focus groups and caution should be exercised when generalising to the broader population.
- As this research was conducted in one country, future research could explore perceptions of the effectiveness of e-cigarette regulations in other jurisdictions.

For peer review only

Introduction

Recent years have seen global increases in the prevalence of e-cigarette use, particularly among youth (Australian Institute of Health and Welfare, 2020; Filippidis, Laverty, Gerovasili, & Vardavas, 2017; Obisesan et al., 2020). In Australia, the context of the present study, the number of adolescents and young adults that report having used an e-cigarette in the past month has increased approximately five-fold since 2018 (Wakefield, Haynes, Tabbakh, Scollo, & Durkin, 2023), with recent figures indicating that 33% of adolescents and young adults have tried e-cigarettes and 14% are regular vapers (i.e., report using e-cigarettes at least monthly) (Pettigrew et al., 2023). These increases in e-cigarette use are concerning given vaping has been found to be associated with several physical and mental health harms and subsequent initiation of tobacco cigarette smoking (Banks et al., 2023; Berry et al., 2019; Lechner, Janssen, Kahler, Audrain-McGovern, & Leventhal, 2017).

The substantial increase in e-cigarette use has prompted calls for tightened regulation of the devices to reduce uptake and minimise potential health risks, especially among youth and those who have never smoked (Jongenelis, Kameron, Rudaizky, & Pettigrew, 2019; World Health Organisation, 2014). In Australia, nicotine-containing e-cigarettes and related products are legally available to adults only via medical prescription (Greenhalgh, Smith, Grace, & Scollo, 2022). E-cigarettes that do not contain nicotine are less restricted and may be sold by retailers to those aged 18+ years in all states and territories except Western Australia (Greenhalgh et al., 2022).

Despite these restrictions, increases in use continue to be observed. This is likely due to (i) a lack of appropriate controls at the Australian border and (ii) the importation and sale of non-nicotine e-cigarette products remaining legal, which has led to mass importation and high availability of non-nicotine and incorrectly-labelled nicotine e-cigarette products on the Australian market (Dessaix, Jardine, Freeman, & Kameron, 2022). To address these issues, Australia's Federal Government announced plans in May 2023 to introduce regulations that prohibit the importation of both nicotine and non-nicotine e-cigarettes for non-therapeutic use (Department of Health and Aged Care, 2023a). Restrictions on flavourings and the introduction of pharmaceutical-like packaging were also proposed to reduce the appeal of e-cigarette products.

For peer review only - http://bmjopen.bmj.com/site/about/guidelines.xhtml

Public acceptability of regulations is an important consideration when developing and implementing policy, with research typically operationalising acceptability as the extent to which a regulation is supported (Diepeveen, Ling, Suhrcke, Roland, & Marteau, 2013; Proctor et al., 2011). Research conducted in Australia has found high levels of support for the introduction of tighter regulations on e-cigarettes, with the vast majority of adults endorsing (i) restricted access to and advertising of e-cigarettes and (ii) the expansion of vape-free public areas (Bain, Mitsopoulos, & Durkin, 2023). Consistent with the notion that support for a particular regulation is moderated by the extent to which that regulation will restrict one's behaviour (Diepeveen et al., 2013), support for e-cigarette regulations has been found to differ based on vaping status. E-cigarette users typically (i) support non-restrictive regulations, such as making e-cigarette products available to all adults via retail stores and allowing vaping in smoke-free areas, and (ii) oppose regulations that restrict the supply of the devices (Fraser, Weier, Keane, & Gartner, 2015; Jongenelis et al., 2019). By contrast, those who have never vaped tend to oppose measures that would result in e-cigarettes being readily available (Jongenelis et al., 2019).

Given support for e-cigarette regulations appears to be heavily moderated by vaping status, considering alternative measures of acceptability is critical to obtaining a more objective account of community views. Perceived effectiveness is one such alternative and an important component of acceptability (Sekhon, Cartwright, & Francis, 2017). Greater perceived effectiveness of a government-initiated health regulation is predictive of more favourable community attitudes toward that regulation and greater compliance (Diepeveen et al., 2013; Smith et al., 2020), suggesting that perceived effectiveness of e-cigarette regulation may provide useful information on the likely outcome of regulation implementation. Despite this, there is a lack of research exploring Australians' perceptions of the effectiveness of current regulations relating to e-cigarettes and their ideas for further regulations that are likely to be effective at reducing e-cigarette use. Accordingly, this study aimed to explore adolescents', young adults', and adults' perspectives on:

1. The effectiveness of current e-cigarette regulations in Australia; and
2. Regulations they believe could minimise e-cigarette use, particularly among adolescents and those who have never smoked.

For peer review only - http://bmjopen.bmj.com/site/about/guidelines.xhtml

Method

Recruitment and Sample

A social research agency was commissioned to recruit a sample of Australians aged 14 to 39 years to participate in one of 16 focus groups conducted in Melbourne and Sydney. Groups were stratified by (i) age (14-15 year olds, 16-17 year olds, 18-24 year olds, and 25-39 year olds), (ii) gender (women and men), and (iii) e-cigarette user status (current/past vapers and those who had never vaped). Table 1 presents the composition of each group. Groups ranged in size from 6 to 10 participants.

For peer review only

Table 1. Focus group characteristics

Group	N	Age	Gender	E-cigarette user status	Location
1	9	14-15 years	Women	Current or past	Sydney
2	10			Never	Melbourne
3	10		Men	Current or past	Sydney
4	10			Never	Melbourne
5	9	16-17 years	Women	Current or past	Melbourne
6	9			Never	Sydney
7	7		Men	Current or past	Melbourne
8	10			Never	Sydney
9	7	18-24 years	Women	Current or past	Melbourne
10	8			Never	Sydney
11	6		Men	Current or past	Melbourne
12	10			Never	Sydney
13	9	25-39 years	Women	Current or past	Melbourne
14	9			Never	Melbourne
15	9		Men	Current or past	Melbourne
16	7			Never	Melbourne

Procedure

Focus groups were conducted in March 2023 (prior to the Government's announcement) and were approximately 70 minutes in duration (range: 57 to 88 minutes). All groups were facilitated by *XX*, a Principal Research Fellow with a PhD in clinical psychology. Participants

For peer review only - http://bmjopen.bmj.com/site/about/guidelines.xhtml

completed a short survey while waiting for their focus group to begin. Items in the survey assessed participants' socio-demographic characteristics (e.g., gender, age).

A semi-structured interview guide comprising open-ended questions was followed. To orient participants to the topics being discussed, all groups began with initial questions exploring participants' experiences with e-cigarettes and knowledge of current regulations. These were then followed by questions exploring (i) participants' opinions of current regulations and (ii) their ideas for what could be done in Australia to effectively reduce e-cigarette use, especially among adolescents and those who have never smoked.

A university Human Research Ethics Committee approved the study (*blinded for review*) and all participants provided written informed consent. For participants aged <16 years, consent was also obtained from a caregiver. Participants were reimbursed AUD120 for their time and the costs associated with focus group attendance. Caregivers were reimbursed AUD30. To ensure adolescents felt comfortable speaking openly about vaping, caregivers were not present during the focus groups.

Patient and Public Involvement

No involvement.

Data Analysis

Focus groups were audio recorded by the research team and transcribed verbatim by an independent and ISO-accredited transcription agency. Transcripts were then imported into NVivo for coding and analysis. As this research was data-driven rather than theory-driven, an inductive approach to thematic analysis was adopted (Braun & Clarke, 2006). One researcher (*XX*) analysed all transcripts according to the iterative steps of the reflexive thematic analysis framework detailed by Braun and Clarke (2019). This included data familiarisation, code generation, theme generation, theme review, defining and naming themes, and combining the analysis of themes in the context of a report (Braun & Clarke, 2019). Regular meetings were held with the facilitator of the focus groups to ensure the coding represented an accurate reflection of the discussions in the groups.

The Matrix Coding Query function in NVivo was used to explore any similarities and differences in data according to (i) age group (adolescents (14-17 year olds) cf. young adults

For peer review only - http://bmjopen.bmj.com/site/about/guidelines.xhtml

(18-24 year olds) cf. adults (25+ year olds)), (ii) gender (women cf. men), and (iii) vaping status (current/past vapers cf. those who had never vaped). Quotes are provided throughout the results section to highlight specific findings of interest. Each participant quote is followed by details of the focus group (FG) of which the participant was a part: FG number (e.g., FG #1); adolescents or young adults or adults aged 25+ years; W = Women or M = Men; V = Vapers or NV = those who had never vaped.

Results

All groups had some experience with e-cigarettes. Among those who had vaped in the past or were current vapers, direct experiences with e-cigarettes were reported. Among those who had never vaped, experiences were indirect and typically involved observing e-cigarette use at school or in the workplace, when out socialising, and by friends or family members. All groups commented that e-cigarette use was increasing and the devices were "everywhere".

Understanding of Australia's current regulations relating to e-cigarettes was low overall, with less than half of all groups mentioning the prescription model for nicotine e-cigarette products. Instead, many groups noted that e-cigarettes were illegal, with some groups specifying that e-cigarettes containing nicotine were illegal. There was a great deal of uncertainty observed, with participants in many groups noting that they were "guessing" when reporting on the regulations. All groups commented on the high availability of e-cigarettes in the community, with most groups reporting that this gave the impression there were no regulations:

> *I feel that there are no regulations in Australia...I feel there is none because of the shops. I also see that there are neon boards with displays at night. You can clearly see that the vape is available. So, everyone has the access to it…it's just like the government is giving them permission to display it.* – FG#14, adults aged 25+ years, W, NV.

Perspectives on Current Regulations

After being informed of the current regulations by the facilitator, groups discussed their perspectives on the prescription model for nicotine e-cigarette products and the availability of non-nicotine e-cigarette products. Half of the groups considered the prescription model to be an effective means of reducing access to e-cigarettes, and thus reducing e-cigarette use. Concerns about the model were raised, however (outlined in detail below). In terms of non-nicotine e-cigarette availability, most groups were unaware that such products existed. When

For peer review only - http://bmjopen.bmj.com/site/about/guidelines.xhtml

informed that these products were available for sale to those aged 18+ years, most groups noted that this availability may increase overall e-cigarette use. Some groups also reported lacking an understanding of why a market would exist for non-nicotine e-cigarettes, noting that *"I don't feel a lot of people would buy non-nicotine ones because they want the nicotine.*" – FG#5, adolescents, W, V.

Perspectives on the availability of nicotine e-cigarette products (i.e., the prescription model)

Half of all groups (predominantly those who had never vaped) considered the prescription model to be effective at reducing e-cigarette use among youth and adults who have never smoked. Participants noted that the model made it difficult for these cohorts to access e-cigarettes:

> *It will stop people being able to buy them, thinking that's okay, and then having access.* – FG#9, young adults, W, V.

Potential issues with the prescription model were raised. In some groups, participants did not believe e-cigarettes were effective smoking cessation aids and reported that the devices have the potential to make people more addicted to nicotine. Accordingly, a medical model was not considered appropriate. Few groups (all of which comprised vapers) discussed potential barriers to accessing a prescription. These barriers included medical practitioner hesitancy to prescribe nicotine e-cigarette products and the financial costs associated with a medical consultation. The prescription model being difficult to enforce and thus easy to circumvent was also raised as a potential issue.

> *I don't think it's going to help you quit. It's just going to make you more addicted to it.* – FG#1, adolescents, F, V.

> *There's such little knowledge about what's actually in these. Why, as a GP, would you recommend that?* – FG#15, adults aged 25+ years, M, V.

> *Who's asking you for a prescription? Is it people in the street who are like, 'Hey, you're smoking that vape, do you have your prescription on you?'* – FG#13, adults aged 25+ years, W, V.

For peer review only - http://bmjopen.bmj.com/site/about/guidelines.xhtml

Perspectives on the availability of non-nicotine e-cigarette products

Opinions on non-nicotine e-cigarette products being available to all adults in Australia were largely negative. Groups voiced concerns that the availability of these products undermines attempts to reduce vaping as these products may be perceived as safe. It was also noted that non-nicotine e-cigarette products lead to addiction and are a gateway to nicotine e-cigarette use. Finally, groups voiced mistrust in the labelling of e-cigarette products given the differing regulations between non-nicotine and nicotine varieties.

> *It's also kind of dumb that they only ban the nicotine one because it makes you think that the other one's safe, when it's probably just as bad."* – FG#3, adolescents, M, V.

> *What's stopping an importer from just putting a sticker that says no nicotine?* – FG#16, adults aged 25+ years, M, NV.

Few positive perspectives on the availability of non-nicotine e-cigarettes were observed. Those voicing a positive perspective (i) noted that non-nicotine e-cigarettes may be used to quit smoking and/or (ii) endorsed libertarianism.

Recommendations for Potential Regulations

When discussing regulatory action that could be taken to effectively reduce e-cigarette use in Australia, groups cited both "demand reduction" and "supply reduction" reforms. Demand reduction reforms were most frequently mentioned, with all groups citing such measures as being important. Perspectives on supply reduction reforms were mixed: groups comprising adolescents, young adults, and those who had never vaped recommended regulations that would heavily restrict e-cigarette availability in the community whereas vapers tended to recommend policies that would increase availability for adults. Suggestions for future regulations were sometimes accompanied by discussions on the addictive nature of e-cigarettes, the link between use and mental health issues, and the importance of ensuring regulations were introduced alongside community- or government-funded support programs. The need for addiction and mental health supports to reduce e-cigarette use were typically raised by adolescents (both vapers and those who had never vaped).

Demand reduction measures

For peer review only - http://bmjopen.bmj.com/site/about/guidelines.xhtml

Specific demand reduction measures cited by focus group participants included introducing plain packaging, including health warnings on e-cigarette products, restricting flavours, increasing the cost of e-cigarette products, increasing the number of vape-free public areas, and restricting advertising. All groups voiced the belief that the introduction of plain packaging and/or health warning messages to mirror existing regulations for tobacco cigarettes would reduce use. Almost all groups reported that prohibiting flavourings and increasing the cost of e-cigarettes would reduce use, particularly among young people. Flavourings were considered highly appealing, and the devices were considered inexpensive and therefore affordable to youth.

They do make vapes look appealing. They're all fluorescent colours, they look good whereas cigarettes are in plain packaging now. I would create consistency across products that, to me, appear more or less the same. – FG#15, adults aged 25+ years, M, V.

If it didn't taste like anything or wasn't nice, if it was yuck, then I feel like it would solve half the problem. – FG#5, adolescents, W, V.

They're [young people] buying a vape now because it's $15 and it's accessible and $15 is nothing. But if you upped that price to $60, people will question if it's worth it. – FG#10, young adults, W, NV.

Most groups discussed increasing the number of vape-free areas and improving signage around vape-free zones. Most groups also voiced concerns that e-cigarettes are marketed towards young people and highly visible in the community due to retail shop advertising. As such, regulations that restrict visibility and advertising were recommended.

Smoke-free zones, or more of them, because they're pretty strong on cigarettes, like 'no smoking in this area', but people are like 'we're going to vape', and it's the norm, but it really shouldn't [be] – FG#11, young adults, M, V.

The outdoor marketing should be banned. There shouldn't be night lights which are clearly saying which shops have access to it… just like all the other cigarette brands that are being sold. – FG#14, adults aged 25+ years, W, NV.

For peer review only - http://bmjopen.bmj.com/site/about/guidelines.xhtml

Supply reduction measures

Perspectives on supply reduction reforms were mixed and appeared to be influenced by factors other than perceived effectiveness, such as libertarian views and participant age and vaping status. Some groups (mostly adolescents and those who had never vaped) believed banning e-cigarettes would be most effective and suggested (i) banning the importation of all e-cigarettes, (ii) banning all disposable e-cigarettes, and (iii) making e-cigarettes less available to children and adolescents. Few groups (mostly vapers) recommended legalising the products in a manner consistent with tobacco cigarettes. Across all groups, the importance of enforcement and taking action against those selling the products illegally was evident:

> *I think just actually enforce the law…If you can just go to any tobacco shop and get it, what even is the point in the law if it's not being enforced…* – FG#8, adolescents, M, NV.

Some differences were observed by age group and vaping status. All adolescent groups discussed supply reduction measures, while half of the young adult and adult groups did so. In terms of vaping status, supply factors were raised by both vapers and those who had never vaped, but those who had never vaped endorsed such measures more strongly. In addition, those who had never vaped typically focused on the importance of reducing use across all age groups (*"I personally think they just shouldn't be allowed.*" – FG#12, young adults, M, NV), whereas vapers focused on reducing use among adolescents (*"They could show IDs and make sure they are 18."* – FG#13, adults aged 25+ years, W, V). Adult and young adult vapers typically discussed a model that mirrors what is currently in place for tobacco cigarettes, with adult access to e-cigarettes legalised. A focus of these discussions was libertarianism, with some participants noting that adults have a right to engage in vaping:

> *Make it legal. I can still go to McDonald's every day and feed my kid McDonald's and it's fine. I'm not going to say that I want to give my kid e-cigs or anything, but as an adult let me adult as long as I'm not hurting someone else.* – FG#15, adults aged 25+ years, M, V.

Finally, some vapers believed that legalisation would reduce e-cigarette use as it would curtail the black market. Some also believed there was hypocrisy in e-cigarettes being less available than cigarettes and noted that there needed to be more evidence on the health impacts of vaping to justify supply restrictions.

For peer review only - http://bmjopen.bmj.com/site/about/guidelines.xhtml

Discussion

E-cigarette use is increasing globally, particularly among adolescents and young people (Australian Institute of Health and Welfare, 2020, Filippidis et al., 2017; Obisesan et al., 2020). This has prompted calls for all levels of government to take regulatory action to minimise uptake of the devices to protect tobacco control efforts (Dessaix et al., 2022; Jongenelis et al., 2019). Given acceptability is an important consideration when implementing new regulations (Proctor et al., 2011), this study examined adolescents', young adults', and adults' (i) perceptions of the effectiveness of Australia's e-cigarette regulations and (ii) recommendations for regulations that have the potential to reduce e-cigarette use, particularly among adolescents and those who have never smoked. Results offer key insights into public perceptions of Australia's regulatory framework for e-cigarette products. They also offer insights for jurisdictions currently considering the implementation of a prescription model (e.g., Scotland) and disposable e-cigarette bans (e.g., the United Kingdom and European Union member states such as Ireland, Germany and France).

Focus group participants lacked a comprehensive understanding of e-cigarette regulations in Australia, with few groups aware of the prescription model for nicotine e-cigarette products. Participants reported that the widespread availability of e-cigarettes and the advertising practices of retail stores that sold e-cigarette products communicated a lack of regulation. Accordingly, the need to enforce existing regulations to improve their effectiveness and reduce the availability of nicotine and non-nicotine e-cigarettes was widely discussed. This was particularly evident when discussing the prescription model for nicotine products, with half of the groups considering the model effective at restricting access to e-cigarettes but only when regulations relating to the model were enforced.

Several other issues relating to the prescription model were raised, albeit by only some groups. These included potential barriers to accessing a prescription, such as cost and practitioner hesitancy to prescribe. Hesitancy among medical practitioners to recommend and/or prescribe e-cigarettes has been identified in previous research (Selamoglu, Erbas, Kasiviswanathan, & Barton, 2022; Stone & Marshall, 2019). Consumers of e-cigarettes have also raised concerns about practitioner hesitancy and the impact of this on access (Jongenelis, Robinson, Hughes, & Pettigrew, 2023). Given the effectiveness of the prescription model is dependent on medical practitioners being open to prescribing e-cigarettes to smokers who wish to quit but have been

For peer review only - http://bmjopen.bmj.com/site/about/guidelines.xhtml

unable to do so with first-line treatments, it is critical that practitioners are supported in their practice to prescribe nicotine e-cigarette products when use is clinically indicated.

Focus group participants were generally unaware of the existence of non-nicotine e-cigarettes and raised several concerns regarding these products when informed of their presence on the Australian market. The most frequently cited concern was that ready availability of these products communicates a message that they are safe. Other concerns included (i) nicotine products being labelled as non-nicotine products to bypass regulations and (ii) non-nicotine product use acting as a gateway to nicotine use. These concerns are supported by the literature. Previous research has found that 60% of e-liquids affixed with a label that claims they are nicotine-free actually contain nicotine (Chivers, Janka, Franklin, Mullins, & Larcombe, 2019). There is also evidence that use of non-nicotine products leads to use of nicotine products, with a recent study finding that approximately 25% of those who exclusively use non-nicotine products will transition to using nicotine products after one year (Nguyen Zarndt, Donaldson, Bernat, Henrie, & Portnoy, 2020). The concerns raised regarding non-nicotine e-cigarettes, and the finding that many in the sample questioned why these products existed and their utility, suggests that plans to restrict access to such products will likely be accepted by the general public.

Concerning potential future regulations, focus group participants recommended several demand and supply reduction measures that they believed would be effective at reducing e-cigarette use. Demand reduction measures were most frequently cited and included placing health warnings on e-cigarette products, restricting flavours, increasing the cost of e-cigarette products, increasing the number of vape-free public areas, and restricting advertising. Given many of these reforms were announced by the Australian Government in May 2023 (Department of Health and Aged Care, 2023a), it can be reasonably expected that implementation of the proposed reforms will be met with little objection from the general public.

Recommended regulations for reducing the supply of e-cigarettes in the community included banning all e-cigarette products, banning the importation of nicotine e-cigarette products, banning all disposable e-cigarettes, reducing access by adolescents, and maintaining adoption of the prescription model. Supply reduction regulations were typically suggested by adolescents and those who had never vaped, whereas adults who vaped tended to adopt a liberal

For peer review only - http://bmjopen.bmj.com/site/about/guidelines.xhtml

approach and recommended models of availability like those in place for tobacco cigarettes. The differing views of adults who vape compared to other participant groups may be explained by perceived threats to existing behaviours or freedoms, and is consistent with previous research showing that e-cigarette users typically oppose regulations that restrict the supply of the devices (Fraser et al., 2015; Jongenelis et al., 2019). Communications highlighting the historical mistake of tobacco cigarettes being made consumer products could be used to inform vapers of the risks associated with regulating e-cigarettes in a manner that is consistent with tobacco products (Jongenelis et al. 2023).

It is encouraging that adolescents and young adults, regardless of vaping status, were largely supportive of a range of demand and supply reduction regulations to address e-cigarette use. These findings suggest Australian youth wish to quit vaping and consider the range of measures proposed, including reduced availability, as critical. This is consistent with other studies that have found high intentions to quit e-cigarettes among young people (Cuccia et al., 2021; Palmer et al., 2022). However, consideration should be given during implementation of supply reduction regulations to ensure appropriate supports are offered to those who are addicted to e-cigarettes or who are using e-cigarettes to cope with mental health struggles, with adolescents in the sample expressing concerns about nicotine dependence and poor mental health.

Limitations and Strengths

This study had some limitations which should be considered when interpreting the findings. First, due to the emergent nature of the coding process, only one researcher coded the data, which prevented the calculation of inter-coder reliability. However, the involvement of the facilitator in the development of the coding hierarchy and refinement of the identified themes enhanced the trustworthiness of the resulting interpretation (Elo et al., 2014; Nowell, Norris, White, & Moules, 2017). Second, the findings only represent the perspectives of the 139 participants who attended the focus groups. Although the size of the sample is large for qualitative research, caution should be exercised when generalising to the broader population. Finally, this research was conducted in one geographic location. Future research could explore perceptions of the effectiveness of e-cigarette regulations in other jurisdictions.

This study had several strengths. To our knowledge this is the first study to explore perceptions of regulation effectiveness, with prior work in this space assessing regulation support. Given support for e-cigarette regulations is influenced by vaping status (Jongenelis et al., 2019), the

For peer review only - http://bmjopen.bmj.com/site/about/guidelines.xhtml

present study's exploration of perceived effectiveness offers a more objective account of community views. Second, we were able to obtain unprompted recommendations for effective e-cigarette regulations prior to the Government's announcement of the new reforms. This provides a real-world opportunity to assess the link between the perceived effectiveness of reforms and the outcomes of implementation. Finally, we recruited participants across the community and stratified groups by age, gender, and vaping status. This allowed us to (i) gather a broader range of perspectives than previous studies and (ii) explore differences between groups. Given participants are more likely to feel comfortable sharing their perspectives in a homogenous group (Krueger, 2014), the stratification also maximised the chances that the discussions held were rich and participant responses were genuine.

Conclusion

Results from the present study exploring perspectives on Australia's e-cigarette regulations and recommendations for further regulations suggest that many believe the current prescription model for nicotine e-cigarette products to be effective when enforced appropriately. Several concerns were raised about the availability of non-nicotine e-cigarettes, suggesting that planned reforms to restrict the supply of these products are likely to be well-received. Given many of the recommendations made by participants for effective regulations were those announced by the Federal Government in the months after this study was conducted, the proposed reforms are likely to be met with little objection from the public. However, ensuring the reforms are complemented by efforts to support those who are addicted to e-cigarettes or who are using e-cigarettes to cope with mental health struggles is critical.

Funding statement: This work was supported by a National Health and Medical Research Council Investigator Grant (APP1194713). The funding source had no involvement in study design; in the collection, analysis and interpretation of data; in the writing of the article; and in the decision to submit the article for publication.

Declarations of interest: None.

Data availability: Upon reasonable request. Data will not be shared with those affiliated with the tobacco or e-cigarette industries.

Author contributions: M.I.J: Funding acquisition, conceptualization, methodology, writing – original manuscript, review and editing, supervision, project administration. S.J.L.Y:

For peer review only - http://bmjopen.bmj.com/site/about/guidelines.xhtml

Methodology, writing – reviewing and editing. M.E.B: Data curation, formal analysis, writing – original manuscript, reviewing, editing.

For peer review only

For peer review only - http://bmjopen.bmj.com/site/about/guidelines.xhtml

References

Australian Institute of Health and Welfare. (2020). National Drug Strategy Household Survey: 2019 - Tobacco Smoking. Canberra: AIHW. Available from: https://www.aihw.gov.au/getmedia/7ebfd47a-9063-4ae0-b22f-1aeff56a30dc/aihw-phe-270-Chapter2-Tobacco.pdf.aspx

Bain, E., Mitsopoulos, E., & Durkin, S. (2023). *Perceptions of and support for policies concerning the availability and use of e-cigarettes among Australian adults, 2021 and 2022.* Retrieved from Melbourne, Australia: https://www.cancervic.org.au/downloads/cbrc/E-cig-policies2023-PUBLIC.pdf

Banks, E., Yazidjoglou, A., Brown, S., Nguyen, M., Martin, M., Beckwith, K., . . . Joshy, G. (2023). Electronic cigarettes and health outcomes: umbrella and systematic review of the global evidence. *Medical Journal of Australia, 218*(6), 267-275. doi:https://doi.org/10.5694/mja2.51890

Berry, K. M., Fetterman, J. L., Benjamin, E. J., Bhatnagar, A., Barrington-Trimis, J. L., Leventhal, A. M., & Stokes, A. (2019). Association of electronic cigarette use with subsequent initiation of tobacco cigarettes in US youths. *JAMA network open, 2*(2), e187794-e187794.

Braun, V., & Clarke, V. (2006). Using thematic analysis in psychology. *Qualitative Research in Psychology, 3*(2), 77-101. doi:10.1191/1478088706qp063oa

Braun, V., & Clarke, V. (2019). Reflecting on reflexive thematic analysis. *Qualitative research in sport, exercise and health, 11*(4), 589-597.

Chivers, E., Janka, M., Franklin, P., Mullins, B., & Larcombe, A. (2019). Nicotine and other potenitally harmful compounds in "nicotine-free" e-cigarette liquids in Australia. *Medical Journal of Australia, 210*(3), 127-128. doi: 10.5694/mja2.12059.

Cuccia, A. F., Patel, M., Amato, M. S., Stephens, D. K., Yoon, S. N., & Vallone, D. M. (2021). Quitting e-cigarettes: Quit attempts and quit intentions among youth and young adults. *Preventive Medicine Reports, 21*, 101287. doi:https://doi.org/10.1016/j.pmedr.2020.101287

For peer review only - http://bmjopen.bmj.com/site/about/guidelines.xhtml

Department of Health and Aged Care. (2023a, May 2). Taking Action on Smoking and Vaping. Available from: https://www.health.gov.au/ministers/the-hon-mark-butler-mp/media/taking-action-on-smoking-and-vaping

Department of Health and Aged Care. (2023b, April 14). About E-cigarettes. Available from: https://www.health.gov.au/topics/smoking-and-tobacco/about-smoking-and-tobacco/about-e-cigarettes

Dessaix, A., Jardine, E., Freeman, B., & Kameron, C. (2022). Undermining Australian controls on electronic nicotine delivery systems: illicit imports and illegal sales. *Tobacco Control, 31*(6), 689-690. doi:10.1136/tc-2022-057772

Diepeveen, S., Ling, T., Suhrcke, M., Roland, M., & Marteau, T. M. (2013). Public acceptability of government intervention to change health-related behaviours: a systematic review and narrative synthesis. *BMC Public Health, 13*(1), 756. doi:10.1186/1471-2458-13-756

Elo, S., Kääriäinen, M., Kanste, O., Pölkki, T., Utriainen, K., & Kyngäs, H. (2014). Qualitative content analysis: A focus on trustworthiness. *SAGE open, 4*(1), 2158244014522633.

Filippidis, F. T., Laverty, A. A., Gerovasili, V., & Vardavas, C. I. (2017). Two-year trends and predictors of e-cigarette use in 27 European Union member states. *Tobacco Control, 26*(1), 98-104. doi:10.1136/tobaccocontrol-2015-052771

Fraser, D., Weier, M., Keane, H., & Gartner, C. (2015). Vapers' perspectives on electronic cigarette regulation in Australia. *International Journal of Drug Policy, 26*(6), 589-594. doi:https://doi.org/10.1016/j.drugpo.2015.01.019

Greenhalgh, E. M., Smith, L., Grace, C., & Scollo, M. (Eds.). (2022). *18.13 E-cigarettes and other alternative nicotine products: Legal status in Australia*. Melbourne: Cancer Council Victoria.

Jongenelis, M. I., Kameron, C., Rudaizky, D., & Pettigrew, S. (2019). Support for e-cigarette regulations among Australian young adults. *BMC Public Health, 19*(1), 67. doi:10.1186/s12889-019-6410-4

For peer review only - http://bmjopen.bmj.com/site/about/guidelines.xhtml

Jongenelis, M. I., Robinson, A., Hughes, A., & Pettigrew, S. (2023). Perceptions of a prescription model for accessing nicotine vaping products: an examination of submissions made by self-reported e-cigarette users to an Australian consultation. *Health Promotion International, 38*(4), daad080.

Krueger, R. A. (2014). *Focus groups: A practical guide for applied research*: Sage publications.

Lechner, W. V., Janssen, T., Kahler, C. W., Audrain-McGovern, J., & Leventhal, A. M. (2017). Bi-directional associations of electronic and combustible cigarette use onset patterns with depressive symptoms in adolescents. *Preventive Medicine, 96*, 73-78.

Nguyen Zarndt, A., Donaldson, E. A., Bernat, J. K., Henrie, J. A., & Portnoy, D. B. (2020). Adult Use of and Transitions From Nicotine and Non-nicotine-Containing E-cigarettes: Data From the Population Assessment of Tobacco and Health (PATH) Study, 2013-2016. *Nicotine Tob Res, 22*(7), 1225-1229. doi:10.1093/ntr/ntz215

Nowell, L. S., Norris, J. M., White, D. E., & Moules, N. J. (2017). Thematic analysis: Striving to meet the trustworthiness criteria. *International journal of qualitative methods, 16*(1), 1609406917733847.

Obisesan, O. H., Osei, A. D., Uddin, S. M. I., Dzaye, O., Mirbolouk, M., Stokes, A., & Blaha, M. J. (2020). Trends in e-Cigarette Use in Adults in the United States, 2016-2018. *JAMA Internal Medicine, 180*(10), 1394-1398. doi:10.1001/jamainternmed.2020.2817

Palmer, A. M., Price, S. N., Foster, M. G., Sanford, B. T., Fucito, L. M., & Toll, B. A. (2022). Urgent Need for Novel Investigations of Treatments to Quit E-cigarettes: Findings from a Systematic Review. *Cancer Prevention Research, 15*(9), 569-580. doi:10.1158/1940-6207.Capr-22-0172

Pettigrew, S., Miller, M., Alvin Santos, J., Raj, T. S., Brown, K., & Jones, A. (2023). E-cigarette attitudes and use in a sample of Australians aged 15–30 years. *Australian and New Zealand Journal of Public Health, 47*(2), 100035. doi:https://doi.org/10.1016/j.anzjph.2023.100035

For peer review only - http://bmjopen.bmj.com/site/about/guidelines.xhtml

Proctor, E., Silmere, H., Raghavan, R., Hovmand, P., Aarons, G., Bunger, A., . . . Hensley, M. (2011). Outcomes for Implementation Research: Conceptual Distinctions, Measurement Challenges, and Research Agenda. *Administration and Policy in Mental Health and Mental Health Services Research, 38*(2), 65-76. doi:10.1007/s10488-010-0319-7

Sekhon, M., Cartwright, M., & Francis, J. J. (2017). Acceptability of healthcare interventions: an overview of reviews and development of a theoretical framework. *BMC Health Services Research, 17*(1), 1-13.

Selamoglu, M., Erbas, B., Kasiviswanathan, K., & Barton, C. (2022). General practitioners' knowledge, attitudes, beliefs and practices surrounding the prescription of e-cigarettes for smoking cessation: a mixed-methods systematic review. *BMC Public Health, 22*(1), 2415. doi:10.1186/s12889-022-14696-3

Smith, L. E., Amlôt, R., Lambert, H., Oliver, I., Robin, C., Yardley, L., & Rubin, G. J. (2020). Factors associated with adherence to self-isolation and lockdown measures in the UK: a cross-sectional survey. *Public Health, 187*, 41-52. doi:https://doi.org/10.1016/j.puhe.2020.07.024

Stone, E., & Marshall, H. (2019). Electronic cigarettes in physician practice: a complex debate. *Internal Medicine Journal, 49*(4), 438-445. doi:https://doi.org/10.1111/imj.14256

Wakefield, M., Haynes, A., Tabbakh, T., Scollo, M., & Durkin, S. (2023). *Current vaping and current smoking in the Australian population ages 14+ years: February 2018-March 2023*. Retrieved from Centre for Beahvioural Research in Cancer, Cancer Council Victoria: Melbourne, Australia:

World Health Organisation. (2014). Backgrounder on WHO report on regulation of e-cigarettes and similar products. *Geneva: Noncommunicable diseases and mental health*.

For peer review only

For peer review only - http://bmjopen.bmj.com/site/about/guidelines.xhtml

Table 1. Focus group characteristics

Group	N	Age	Gender	E-cigarette user status	Location
1	9	14-15 years	Women	Current or past	Sydney
2	10			Never	Melbourne
3	10		Men	Current or past	Sydney
4	10			Never	Melbourne
5	9	16-17 years	Women	Current or past	Melbourne
6	9			Never	Sydney
7	7		Men	Current or past	Melbourne
8	10			Never	Sydney
9	7	18-24 years	Women	Current or past	Melbourne
10	8			Never	Sydney
11	6		Men	Current or past	Melbourne
12	10			Never	Sydney
13	9	25-39 years	Women	Current or past	Melbourne
14	9			Never	Melbourne
15	9		Men	Current or past	Melbourne
16	7			Never	Melbourne

For peer review only

Standards for Reporting Qualitative Research (SRQR)*

http://www.equator-network.org/reporting-guidelines/srqr/

Page/line no(s).

Title and abstract

**Title** - Concise description of the nature and topic of the study Identifying the study as qualitative or indicating the approach (e.g., ethnography, grounded theory) or data collection methods (e.g., interview, focus group) is recommended	Pages 1 and 2
**Abstract** - Summary of key elements of the study using the abstract format of the intended publication; typically includes background, purpose, methods, results, and conclusions	Page 2

Introduction

**Problem formulation** - Description and significance of the problem/phenomenon studied; review of relevant theory and empirical work; problem statement	Page 4 and 5
**Purpose or research question** - Purpose of the study and specific objectives or questions	Page 5

Methods

**Qualitative approach and research paradigm** - Qualitative approach (e.g., ethnography, grounded theory, case study, phenomenology, narrative research) and guiding theory if appropriate; identifying the research paradigm (e.g., postpositivist, constructivist/ interpretivist) is also recommended; rationale**	Page 7
**Researcher characteristics and reflexivity** - Researchers' characteristics that may influence the research, including personal attributes, qualifications/experience, relationship with participants, assumptions, and/or presuppositions; potential or actual interaction between researchers' characteristics and the research questions, approach, methods, results, and/or transferability	Pages 6 and 7
**Context** - Setting/site and salient contextual factors; rationale**	Pages 5-7
**Sampling strategy** - How and why research participants, documents, or events were selected; criteria for deciding when no further sampling was necessary (e.g., sampling saturation); rationale**	Page 6
**Ethical issues pertaining to human subjects** - Documentation of approval by an appropriate ethics review board and participant consent, or explanation for lack thereof; other confidentiality and data security issues	Page 7
**Data collection methods** - Types of data collected; details of data collection procedures including (as appropriate) start and stop dates of data collection and analysis, iterative process, triangulation of sources/methods, and modification of procedures in response to evolving study findings; rationale**	Pages 6 and 7

For peer review only

For peer review only - http://bmjopen.bmj.com/site/about/guidelines.xhtml

**Data collection instruments and technologies** - Description of instruments (e.g., interview guides, questionnaires) and devices (e.g., audio recorders) used for data collection; if/how the instrument(s) changed over the course of the study	Pages 6 and 7
**Units of study** - Number and relevant characteristics of participants, documents, or events included in the study; level of participation (could be reported in results)	Pages 6 and 7
**Data processing** - Methods for processing data prior to and during analysis, including transcription, data entry, data management and security, verification of data integrity, data coding, and anonymization/de-identification of excerpts	Page 7
**Data analysis** - Process by which inferences, themes, etc., were identified and developed, including the researchers involved in data analysis; usually references a specific paradigm or approach; rationale**	Page 7
**Techniques to enhance trustworthiness** - Techniques to enhance trustworthiness and credibility of data analysis (e.g., member checking, audit trail, triangulation); rationale**	Page 7

Results/findings

**Synthesis and interpretation** - Main findings (e.g., interpretations, inferences, and themes); might include development of a theory or model, or integration with prior research or theory	Pages 8-12
**Links to empirical data** - Evidence (e.g., quotes, field notes, text excerpts, photographs) to substantiate analytic findings	Page 8-12

Discussion

**Integration with prior work, implications, transferability, and contribution(s) to the field -** Short summary of main findings; explanation of how findings and conclusions connect to, support, elaborate on, or challenge conclusions of earlier scholarship; discussion of scope of application/generalizability; identification of unique contribution(s) to scholarship in a discipline or field	Pages 12-16
**Limitations** - Trustworthiness and limitations of findings	Page 15

Other

**Conflicts of interest** - Potential sources of influence or perceived influence on study conduct and conclusions; how these were managed	Page 16
**Funding** - Sources of funding and other support; role of funders in data collection, interpretation, and reporting	Page 16

*The authors created the SRQR by searching the literature to identify guidelines, reporting standards, and critical appraisal criteria for qualitative research; reviewing the reference lists of retrieved sources; and contacting experts to gain feedback. The SRQR aims to improve the transparency of all aspects of qualitative research by providing clear standards for reporting qualitative research.

For peer review only

For peer review only - http://bmjopen.bmj.com/site/about/guidelines.xhtml

**The rationale should briefly discuss the justification for choosing that theory, approach, method, or technique rather than other options available, the assumptions and limitations implicit in those choices, and how those choices influence study conclusions and transferability. As appropriate, the rationale for several items might be discussed together.

Reference:

O'Brien BC, Harris IB, Beckman TJ, Reed DA, Cook DA. **Standards for reporting qualitative research: a synthesis of recommendations.** *Academic Medicine*, Vol. 89, No. 9 / Sept 2014 DOI: 10.1097/ACM.0000000000000388

For peer review only

BMJ Open

Perceptions of Australia's e-cigarette regulations and recommendations for future reforms: A qualitative study of adolescents and adults

Journal:	*BMJ Open*
Manuscript ID	bmjopen-2023-081032.R1
Article Type:	Original research
Date Submitted by the Author:	17-Jan-2024
Complete List of Authors:	Brierley, Mary-Ellen; The University of Melbourne, School of Psychological Sciences<br>Yaw, Sean; The University of Melbourne; The University of Melbourne<br>Jongenelis, Michelle I. ; The University of Melbourne
**Primary Subject Heading**:	Public health
Secondary Subject Heading:	Smoking and tobacco
Keywords:	PUBLIC HEALTH, Health policy < HEALTH SERVICES ADMINISTRATION & MANAGEMENT, Adolescents < Adolescent, QUALITATIVE RESEARCH

SCHOLARONE™
Manuscripts

I, the Submitting Author has the right to grant and does grant on behalf of all authors of the Work (as defined in the below author licence), an exclusive licence and/or a non-exclusive licence for contributions from authors who are: i) UK Crown employees; ii) where BMJ has agreed a CC-BY licence shall apply, and/or iii) in accordance with the terms applicable for US Federal Government officers or employees acting as part of their official duties; on a worldwide, perpetual, irrevocable, royalty-free basis to BMJ Publishing Group Ltd ("BMJ") its licensees and where the relevant Journal is co-owned by BMJ to the co-owners of the Journal, to publish the Work in this journal and any other BMJ products and to exploit all rights, as set out in our licence.

The Submitting Author accepts and understands that any supply made under these terms is made by BMJ to the Submitting Author unless you are acting as an employee on behalf of your employer or a postgraduate student of an affiliated institution which is paying any applicable article publishing charge ("APC") for Open Access articles. Where the Submitting Author wishes to make the Work available on an Open Access basis (and intends to pay the relevant APC), the terms of reuse of such Open Access shall be governed by a Creative Commons licence – details of these licences and which Creative Commons licence will apply to this Work are set out in our licence referred to above.

Other than as permitted in any relevant BMJ Author's Self Archiving Policies, I confirm this Work has not been accepted for publication elsewhere, is not being considered for publication elsewhere and does not duplicate material already published. I confirm all authors consent to publication of this Work and authorise the granting of this licence.

For peer review only

Perceptions of Australia's e-cigarette regulations and recommendations for future reforms: A qualitative study of adolescents and adults

Mary-Ellen E. Brierley[a] , Sean J. L. Yaw[a,b], and Michelle I. Jongenelis[a*]

[a]Melbourne Centre for Behaviour Change, Melbourne School of Psychological Sciences,
The University of Melbourne,
Grattan Street, Parkville, 3010
Australia
[b]Melbourne School of Population and Global Health, The University of Melbourne,
Grattan Street, Parkville, 3010
Australia

*CORRESPONDING AUTHOR
Michelle.jongenelis@unimelb.edu.au
+61 03 9035 4979
ORCID: 0000-0002-0717-1692

Word count
Abstract: 251
Main manuscript: 4394

For peer review only - http://bmjopen.bmj.com/site/about/guidelines.xhtml

Perceptions of Australia's e-cigarette regulations and recommendations for future reforms: A qualitative study of adolescents and adults

Abstract

Objective: To assess public perceptions of the effectiveness of e-cigarette regulations in minimising use among adolescents and those who have never smoked. Specifically, we explored (i) perceived effectiveness of current regulations relating to e-cigarettes and (ii) ideas for further regulations that could reduce use.

Design and participants: Focus groups (n = 16) were conducted with Australian adolescents (14 to 17 year olds), young adults (18 to 24 year olds), and adults (25 to 39 year olds). Groups were stratified by age, gender, and e-cigarette use status. Data were analysed using reflexive thematic analysis.

Setting: Focus groups were conducted in-person in two major Australian cities.

Results: Groups lacked a comprehensive understanding of e-cigarette regulations in Australia. When informed of these regulations, half of the groups considered the prescription model for nicotine e-cigarette products to be effective when enforced appropriately. Almost all groups considered adult access to non-nicotine products problematic. All groups suggested a range of demand reduction regulations, including plain packaging, health warnings, flavour restrictions, and increased vape-free areas. Most groups (predominantly those who had never vaped) also recommended supply reduction regulations such as banning all e-cigarettes. The need for supply reduction measures to include addiction and mental health supports was discussed.

Conclusions: The regulations recommended by participants largely align with those that are to be introduced in Australia, indicating that these reforms are likely to be accepted by the public. Ensuring these reforms are complemented by formal supports for young people experiencing nicotine dependence and related mental health concerns is critical.

Keywords: e-cigarettes; regulations; policy; perceived effectiveness; nicotine; adolescents; young adults; adults.

For peer review only

Article summary: Strengths and limitations of this study

- This was the first study to explore perceptions of e-cigarette regulation effectiveness, with prior work in this space assessing regulation support only.
- We recruited participants across the community and stratified groups by age, gender, and vaping status to gather a broad range of perspectives and explore differences between groups.
- Due to the emergent nature of the coding process, only one researcher coded the data, which prevented the calculation of inter-coder reliability.
- Findings only represent the perspectives of the 139 participants who attended the focus groups and caution should be exercised when generalising to the broader population.
- As this research was conducted in one country, future research could explore perceptions of the effectiveness of e-cigarette regulations in other jurisdictions.

For peer review only

For peer review only - http://bmjopen.bmj.com/site/about/guidelines.xhtml

Introduction

Recent years have seen global increases in the prevalence of e-cigarette use, particularly among youth [1, 2]. In Australia, the context of the present study, e-cigarette use has increased significantly since reporting measures were introduced in 2013 [3], with the number of adolescents and young adults that report having used an e-cigarette in the past month increasing approximately five-fold since 2018 [4]. These increases are concerning given vaping has been found to be associated with several physical and mental health harms and subsequent initiation of tobacco cigarette smoking [5-7]. The increasing prevalence of e-cigarette use has largely occurred in the context of decreasing tobacco cigarette use, although recent data has observed an increase in tobacco smoking among adolescents and young adults for the first time in three decades [4].

The substantial increase in e-cigarette use has prompted calls for tightened regulation of the devices to reduce uptake and minimise potential health risks, especially among youth and those who have never smoked [8, 9]. Since the 1st October 2021, nicotine-containing e-cigarettes and related products have been legally available to adults only via prescription from a medical doctor for the purposes of smoking cessation [10]. E-cigarettes that do not contain nicotine are less restricted and may be sold by retailers to those aged 18+ years in all states and territories except Western Australia [10]. The supply of e-cigarettes – regardless of nicotine content – to individuals under 18 years of age has never been permitted.

Despite these restrictions, increases in use continue to be observed. This is likely due to (i) a lack of appropriate controls at the Australian border and (ii) the importation and sale of non-nicotine e-cigarette products remaining legal, which has led to mass importation and high availability of non-nicotine and incorrectly-labelled nicotine e-cigarette products on the Australian market [11]. To address these issues, Australia's Federal Government announced plans in May 2023 to introduce regulations that prohibit the importation of both nicotine and non-nicotine e-cigarettes for non-therapeutic use [12]. Restrictions on flavourings and the introduction of pharmaceutical-like packaging were also proposed to reduce the appeal of e-cigarette products. These regulations will be implemented on the 1st March 2024 [13].

Public acceptability of regulations is an important consideration when developing and implementing policy, with research typically operationalising acceptability as the extent to which a regulation is supported [14, 15]. Research conducted in Australia has found high levels

For peer review only

of support for the introduction of tighter regulations on e-cigarettes, with the vast majority of adults endorsing (i) restricted access to and advertising of e-cigarettes and (ii) the expansion of vape-free public areas [16]. Consistent with the notion that support for a particular regulation is moderated by the extent to which that regulation will restrict one's behaviour [14], support for e-cigarette regulations has been found to differ based on vaping status. E-cigarette users typically (i) support non-restrictive regulations, such as making e-cigarette products available to all adults via retail stores and allowing vaping in smoke-free areas, and (ii) oppose regulations that restrict the supply of the devices [9, 17]. By contrast, those who have never vaped tend to oppose measures that would result in e-cigarettes being readily available [9].

Given support for e-cigarette regulations appears to be heavily moderated by vaping status, considering alternative measures of acceptability is critical to obtaining a more objective account of community views. Perceived effectiveness is one such alternative and an important component of acceptability [18]. Greater perceived effectiveness of a government-initiated health regulation is predictive of more favourable community attitudes toward that regulation and greater compliance [14, 19], suggesting that perceived effectiveness of e-cigarette regulation may provide useful information on the likely outcome of regulation implementation. Despite this, there is a lack of research exploring Australians' perceptions of the effectiveness of current regulations relating to e-cigarettes and their ideas for further regulations that are likely to be effective at reducing e-cigarette use. Accordingly, this study aimed to explore adolescents', young adults', and adults' perspectives on:

1. The effectiveness of current e-cigarette regulations in Australia; and
2. Regulations they believe could minimise e-cigarette use, particularly among adolescents and those who have never smoked.

Method

Recruitment and Sample

A social research agency was commissioned to recruit a sample of Australians aged 14 to 39 years to participate in one of 16 focus groups conducted in Melbourne and Sydney. Groups were stratified by (i) age (14-15 year olds, 16-17 year olds, 18-24 year olds, and 25-39 year olds), (ii) gender (women and men), and (iii) e-cigarette user status (current/past vapers and

For peer review only - http://bmjopen.bmj.com/site/about/guidelines.xhtml

those who had never vaped). Table 1 presents the composition of each group. Groups ranged in size from 6 to 10 participants.

Table 1. Focus group characteristics

Group	N	Age	Gender	E-cigarette user status	Location
1	9	14-15 years	Women	Current or past	Sydney
2	10			Never	Melbourne
3	10		Men	Current or past	Sydney
4	10			Never	Melbourne
5	9	16-17 years	Women	Current or past	Melbourne
6	9			Never	Sydney
7	7		Men	Current or past	Melbourne
8	10			Never	Sydney
9	7	18-24 years	Women	Current or past	Melbourne
10	8			Never	Sydney
11	6		Men	Current or past	Melbourne
12	10			Never	Sydney
13	9	25-39 years	Women	Current or past	Melbourne
14	9			Never	Melbourne
15	9		Men	Current or past	Melbourne
16	7			Never	Melbourne

For peer review only

Procedure

Focus groups were conducted in March 2023 (prior to the Government's announcement) and were approximately 70 minutes in duration (range: 57 to 88 minutes). All groups were facilitated by *XX*, a Principal Research Fellow with a PhD in clinical psychology. Participants completed a short survey while waiting for their focus group to begin. Items in the survey assessed participants' socio-demographic characteristics (e.g., gender, age).

A semi-structured interview guide comprising open-ended questions was followed. To orient participants to the topics being discussed, all groups began with initial questions exploring participants' experiences with e-cigarettes and knowledge of current regulations. These were

For peer review only - http://bmjopen.bmj.com/site/about/guidelines.xhtml

then followed by questions exploring (i) participants' opinions of current regulations and (ii) their ideas for what could be done in Australia to effectively reduce e-cigarette use, especially among adolescents and those who have never smoked.

A university Human Research Ethics Committee approved the study (*blinded for review*) and all participants provided written informed consent. For participants aged <16 years, consent was also obtained from a caregiver. Participants were reimbursed AUD120 for their time and the costs associated with focus group attendance. Caregivers were reimbursed AUD30. To ensure adolescents felt comfortable speaking openly about vaping, caregivers were not present during the focus groups.

Patient and Public Involvement

No involvement.

Data Analysis

Focus groups were audio recorded by the research team and transcribed verbatim by an independent and ISO-accredited transcription agency. Transcripts were then imported into NVivo for coding and analysis. As this research was data-driven rather than theory-driven, an inductive (i.e., emergent) approach to thematic analysis was adopted [20]. One researcher (*YY*) analysed all transcripts according to the iterative steps of the reflexive thematic analysis framework detailed by Braun and Clarke [21]. This included data familiarisation, code generation, theme generation, theme review, defining and naming themes, and combining the analysis of themes in the context of a report [21]. Throughout this process, a series of 'critical friends' meetings was held [22]. During these meetings, XX and YY reviewed the coding hierarchy, refined the identified codes, and generated key themes based on these codes.

Initial codes were generated that organised the data into the following broad topics: (i) beliefs about current regulations and (ii) recommendations for potential regulations. For the former, child codes were created that further organised the data into the topics of (i) beliefs about the prescription model and (ii) beliefs about the availability of non-nicotine e-cigarette products. Within each of these child codes, further codes were generated that organised the data based on (i) positive sentiment and (ii) negative sentiment.

For peer review only - http://bmjopen.bmj.com/site/about/guidelines.xhtml

In terms of regulation recommendations, child codes were created for each of the ideas generated by participants (e.g., price increases, plain packaging, product warning labels). Codes featuring similar content were then merged (e.g., 'plain packaging' and 'product warning labels' were merged to form a 'product packaging' code). These codes were then refined into the categories of (i) supply factors and (ii) demand factors. Descriptive labels were assigned to all codes. Please see supplementary material for the final coding hierarchy.

The Matrix Coding Query function in NVivo was used to explore any similarities and differences in data according to (i) age group (adolescents (14-17 year olds) cf. young adults (18-24 year olds) cf. adults (25+ year olds)), (ii) gender (women cf. men), and (iii) vaping status (current/past vapers cf. those who had never vaped). Quotes are provided throughout the results section to highlight specific findings of interest. Each participant quote is followed by details of the focus group (FG) of which the participant was a part: FG number (e.g., FG #1); adolescents or young adults or adults aged 25+ years; W = Women or M = Men; V = Vapers or NV = those who had never vaped.

Results

All groups had some experience with e-cigarettes. Among those who had vaped in the past or were current vapers, direct experiences with e-cigarettes were reported. Among those who had never vaped, experiences were indirect and typically involved observing e-cigarette use at school or in the workplace, when out socialising, and by friends or family members. All groups commented that e-cigarette use was increasing and the devices were "everywhere".

Understanding of Australia's current regulations relating to e-cigarettes was low overall, with less than half of all groups mentioning the prescription model for nicotine e-cigarette products. Instead, many groups noted that e-cigarettes were illegal, with some groups specifying that e-cigarettes containing nicotine were illegal. There was a great deal of uncertainty observed, with participants in many groups noting that they were "guessing" when reporting on the regulations. All groups commented on the high availability of e-cigarettes in the community, with most groups reporting that this gave the impression there were no regulations:

I feel that there are no regulations in Australia...I feel there is none because of the shops. I also see that there are neon boards with displays at night. You can clearly see that the vape

For peer review only - http://bmjopen.bmj.com/site/about/guidelines.xhtml

is available. So, everyone has the access to it…it's just like the government is giving them permission to display it. – FG#14, adults aged 25+ years, W, NV.

Perspectives on Current Regulations

After being informed of the current regulations by the facilitator, groups discussed their perspectives on the prescription model for nicotine e-cigarette products and the availability of non-nicotine e-cigarette products. Half of the groups considered the prescription model to be an effective means of reducing access to e-cigarettes, and thus reducing e-cigarette use. Concerns about the model were raised, however (outlined in detail below). In terms of non-nicotine e-cigarette availability, most groups were unaware that such products existed. When informed that these products were available for sale to those aged 18+ years, most groups noted that this availability may increase overall e-cigarette use. Some groups also reported lacking an understanding of why a market would exist for non-nicotine e-cigarettes, noting that *"I don't feel a lot of people would buy non-nicotine ones because they want the nicotine.*" – FG#5, adolescents, W, V.

Perspectives on the availability of nicotine e-cigarette products (i.e., the prescription model)

Half of all groups (predominantly those who had never vaped) considered the prescription model to be effective at reducing e-cigarette use among youth and adults who have never smoked. Participants noted that the model made it difficult for these cohorts to access e-cigarettes:

It will stop people being able to buy them, thinking that's okay, and then having access. – FG#9, young adults, W, V.

Potential issues with the prescription model were raised. In some groups, participants did not believe e-cigarettes were effective smoking cessation aids and reported that the devices have the potential to make people more addicted to nicotine. Accordingly, a medical model was not considered appropriate. Few groups (all of which comprised vapers) discussed potential barriers to accessing a prescription. These barriers included medical practitioner hesitancy to prescribe nicotine e-cigarette products and the financial costs associated with a medical consultation. The prescription model being difficult to enforce and thus easy to circumvent was also raised as a potential issue.

For peer review only - http://bmjopen.bmj.com/site/about/guidelines.xhtml

I don't think it's going to help you quit. It's just going to make you more addicted to it. – FG#1, adolescents, F, V.

There's such little knowledge about what's actually in these. Why, as a GP, would you recommend that? – FG#15, adults aged 25+ years, M, V.

Who's asking you for a prescription? Is it people in the street who are like, 'Hey, you're smoking that vape, do you have your prescription on you?' – FG#13, adults aged 25+ years, W, V.

Perspectives on the availability of non-nicotine e-cigarette products

Opinions on non-nicotine e-cigarette products being available to all adults in Australia were largely negative. Groups voiced concerns that the availability of these products undermines attempts to reduce vaping as these products may be perceived as safe. It was also noted that non-nicotine e-cigarette products lead to addiction and are a gateway to nicotine e-cigarette use. Finally, groups voiced mistrust in the labelling of e-cigarette products given the differing regulations between non-nicotine and nicotine varieties.

It's also kind of dumb that they only ban the nicotine one because it makes you think that the other one's safe, when it's probably just as bad." – FG#3, adolescents, M, V.

What's stopping an importer from just putting a sticker that says no nicotine? – FG#16, adults aged 25+ years, M, NV.

Few positive perspectives on the availability of non-nicotine e-cigarettes were observed. Those voicing a positive perspective (i) noted that non-nicotine e-cigarettes may be used to quit smoking and/or (ii) endorsed libertarianism.

Recommendations for Potential Regulations

When discussing regulatory action that could be taken to effectively reduce e-cigarette use in Australia, groups cited both "demand reduction" and "supply reduction" reforms. Demand reduction reforms were most frequently mentioned, with all groups citing such measures as being important. Perspectives on supply reduction reforms were mixed: groups comprising adolescents, young adults, and those who had never vaped recommended regulations that

For peer review only - http://bmjopen.bmj.com/site/about/guidelines.xhtml

would heavily restrict e-cigarette availability in the community whereas vapers tended to recommend policies that would increase availability for adults. Suggestions for future regulations were sometimes accompanied by discussions on the addictive nature of e-cigarettes, the link between use and mental health issues, and the importance of ensuring regulations were introduced alongside community- or government-funded support programs. The need for addiction and mental health supports to reduce e-cigarette use were typically raised by adolescents (both vapers and those who had never vaped).

Demand reduction measures

Specific demand reduction measures cited by focus group participants included introducing plain packaging, including health warnings on e-cigarette products, restricting flavours, increasing the cost of e-cigarette products, increasing the number of vape-free public areas, and restricting advertising. All groups voiced the belief that the introduction of plain packaging and/or health warning messages to mirror existing regulations for tobacco cigarettes would reduce use. Almost all groups reported that prohibiting flavourings and increasing the cost of e-cigarettes would reduce use, particularly among young people. Flavourings were considered highly appealing, and the devices were considered inexpensive and therefore affordable to youth.

> *They do make vapes look appealing. They're all fluorescent colours, they look good whereas cigarettes are in plain packaging now. I would create consistency across products that, to me, appear more or less the same.* – FG#15, adults aged 25+ years, M, V.

> *If it didn't taste like anything or wasn't nice, if it was yuck, then I feel like it would solve half the problem.* – FG#5, adolescents, W, V.

> *They're [young people] buying a vape now because it's $15 and it's accessible and $15 is nothing. But if you upped that price to $60, people will question if it's worth it.* – FG#10, young adults, W, NV.

Most groups discussed increasing the number of vape-free areas and improving signage around vape-free zones. Most groups also voiced concerns that e-cigarettes are marketed towards young people and highly visible in the community due to retail shop advertising. As such, regulations that restrict visibility and advertising were recommended.

For peer review only - http://bmjopen.bmj.com/site/about/guidelines.xhtml

For peer review only

Smoke-free zones, or more of them, because they're pretty strong on cigarettes, like 'no smoking in this area', but people are like 'we're going to vape', and it's the norm, but it really shouldn't [be] – FG#11, young adults, M, V.

The outdoor marketing should be banned. There shouldn't be night lights which are clearly saying which shops have access to it… just like all the other cigarette brands that are being sold. – FG#14, adults aged 25+ years, W, NV.

Supply reduction measures

Perspectives on supply reduction reforms were mixed and appeared to be influenced by factors other than perceived effectiveness, such as libertarian views and participant age and vaping status. Some groups (mostly adolescents and those who had never vaped) believed banning e-cigarettes would be most effective and suggested (i) banning the importation of all e-cigarettes, (ii) banning all disposable e-cigarettes, and (iii) making e-cigarettes less available to children and adolescents. Few groups (mostly vapers) recommended legalising the products in a manner consistent with tobacco cigarettes. Across all groups, the importance of enforcement and taking action against those selling the products illegally was evident:

I think just actually enforce the law…If you can just go to any tobacco shop and get it, what even is the point in the law if it's not being enforced… – FG#8, adolescents, M, NV.

Some differences were observed by age group and vaping status. All adolescent groups discussed supply reduction measures, while half of the young adult and adult groups did so. In terms of vaping status, supply factors were raised by both vapers and those who had never vaped, but those who had never vaped endorsed such measures more strongly. In addition, those who had never vaped typically focused on the importance of reducing use across all age groups (*"I personally think they just shouldn't be allowed.*" – FG#12, young adults, M, NV), whereas vapers focused on reducing use among adolescents (*"They could show IDs and make sure they are 18."* – FG#13, adults aged 25+ years, W, V). Adult and young adult vapers typically discussed a model that mirrors what is currently in place for tobacco cigarettes, with adult access to e-cigarettes legalised. A focus of these discussions was libertarianism, with some participants noting that adults have a right to engage in vaping:

For peer review only - http://bmjopen.bmj.com/site/about/guidelines.xhtml

Make it legal. I can still go to McDonald's every day and feed my kid McDonald's and it's fine. I'm not going to say that I want to give my kid e-cigs or anything, but as an adult let me adult as long as I'm not hurting someone else. – FG#15, adults aged 25+ years, M, V.

Finally, some vapers believed that legalisation would reduce e-cigarette use as it would curtail the black market. Some also believed there was hypocrisy in e-cigarettes being less available than cigarettes and noted that there needed to be more evidence on the health impacts of vaping to justify supply restrictions.

Discussion

E-cigarette use is increasing globally, particularly among adolescents and young people [1, 2]. This has prompted calls for all levels of government to take regulatory action to minimise uptake of the devices to protect tobacco control efforts [9, 11]. Given acceptability is an important consideration when implementing new regulations [15], this study examined adolescents', young adults', and adults' (i) perceptions of the effectiveness of Australia's e-cigarette regulations and (ii) recommendations for regulations that have the potential to reduce e-cigarette use, particularly among adolescents and those who have never smoked. Results offer key insights into public perceptions of Australia's regulatory framework for e-cigarette products. They also offer insights for jurisdictions currently considering the implementation of a prescription model (e.g., Scotland) and disposable e-cigarette bans (e.g., the United Kingdom and European Union member states such as Ireland, Germany and France).

Focus group participants lacked a comprehensive understanding of e-cigarette regulations in Australia, with few groups aware of the prescription model for nicotine e-cigarette products. Participants reported that the widespread availability of e-cigarettes and the advertising practices of retail stores that sold e-cigarette products communicated a lack of regulation. Accordingly, the need to enforce existing regulations to improve their effectiveness and reduce the availability of nicotine and non-nicotine e-cigarettes was widely discussed. This was particularly evident when discussing the prescription model for nicotine products, with half of the groups considering the model effective at restricting access to e-cigarettes but only when regulations relating to the model were enforced.

Several other issues relating to the prescription model were raised, albeit by only some groups. These included potential barriers to accessing a prescription, such as cost and practitioner

For peer review only - http://bmjopen.bmj.com/site/about/guidelines.xhtml

For peer review only

hesitancy to prescribe. Hesitancy among medical practitioners to recommend and/or prescribe e-cigarettes has been identified in previous research [23, 24]. Consumers of e-cigarettes have also raised concerns about practitioner hesitancy and the impact of this on access [25]. Given the effectiveness of the prescription model is dependent on medical practitioners being open to prescribing e-cigarettes to smokers who wish to quit but have been unable to do so with first-line treatments, it is critical that practitioners are supported in their practice to prescribe nicotine e-cigarette products when use is clinically indicated.

Focus group participants were generally unaware of the existence of non-nicotine e-cigarettes and raised several concerns regarding these products when informed of their presence on the Australian market. The most frequently cited concern was that ready availability of these products communicates a message that they are safe. Other concerns included (i) nicotine products being labelled as non-nicotine products to bypass regulations and (ii) non-nicotine product use acting as a gateway to nicotine use. These concerns are supported by the literature. Previous research has found that 60% of e-liquids affixed with a label that claims they are nicotine-free actually contain nicotine [26]. There is also evidence that use of non-nicotine products leads to use of nicotine products, with a recent study finding that approximately 25% of those who exclusively use non-nicotine products will transition to using nicotine products after one year [27]. The concerns raised regarding non-nicotine e-cigarettes, and the finding that many in the sample questioned why these products existed and their utility, suggests that plans to restrict access to such products will likely be accepted by the general public.

Concerning potential future regulations, focus group participants recommended several demand and supply reduction measures that they believed would be effective at reducing e-cigarette use. Demand reduction measures were most frequently cited and included placing health warnings on e-cigarette products, restricting flavours, increasing the cost of e-cigarette products, increasing the number of vape-free public areas, and restricting advertising. Given many of these reforms were announced by the Australian Government in May 2023 [12], it can be reasonably expected that implementation of the proposed reforms will be met with little objection from the general public.

Recommended regulations for reducing the supply of e-cigarettes in the community included banning all e-cigarette products, banning the importation of nicotine e-cigarette products, banning all disposable e-cigarettes, reducing access by adolescents, and maintaining adoption

For peer review only - http://bmjopen.bmj.com/site/about/guidelines.xhtml

of the prescription model. Supply reduction regulations were typically suggested by adolescents and those who had never vaped, whereas adults who vaped tended to adopt a liberal approach and recommended models of availability like those in place for tobacco cigarettes. The differing views of adults who vape compared to other participant groups may be explained by perceived threats to existing behaviours or freedoms, and is consistent with previous research showing that e-cigarette users typically oppose regulations that restrict the supply of the devices [9, 17]. Communications highlighting the historical mistake of tobacco cigarettes being made consumer products could be used to inform vapers of the risks associated with regulating e-cigarettes in a manner that is consistent with tobacco products [25].

It is encouraging that adolescents and young adults, regardless of vaping status, were largely supportive of a range of demand and supply reduction regulations to address e-cigarette use. These findings suggest Australian youth wish to quit vaping and consider the range of measures proposed, including reduced availability, as critical. This is consistent with other studies that have found high intentions to quit e-cigarettes among young people [28, 29]. However, consideration should be given during implementation of supply reduction regulations to ensure appropriate supports are offered to those who are addicted to e-cigarettes or who are using e-cigarettes to cope with mental health struggles, with adolescents in the sample expressing concerns about nicotine dependence and poor mental health.

Limitations and Strengths

This study had some limitations which should be considered when interpreting the findings. First, due to the emergent nature of the coding process, only one researcher coded the data, which prevented the calculation of inter-coder reliability. However, the involvement of the facilitator in the development of the coding hierarchy and refinement of the identified themes enhanced the trustworthiness of the resulting interpretation [30, 31]. Second, the findings only represent the perspectives of the 139 participants who attended the focus groups. Although the size of the sample is large for qualitative research, caution should be exercised when generalising to the broader population. Finally, this research was conducted in one geographic location. Future research could explore perceptions of the effectiveness of e-cigarette regulations in other jurisdictions.

This study had several strengths. To our knowledge this is the first study to explore perceptions of regulation effectiveness, with prior work in this space assessing regulation support. Given

For peer review only - http://bmjopen.bmj.com/site/about/guidelines.xhtml

support for e-cigarette regulations is influenced by vaping status [9], the present study's exploration of perceived effectiveness offers a more objective account of community views. Second, we were able to obtain unprompted recommendations for effective e-cigarette regulations prior to the Government's announcement of the new reforms. This provides a real-world opportunity to assess the link between the perceived effectiveness of reforms and the outcomes of implementation. Finally, we recruited participants across the community and stratified groups by age, gender, and vaping status. This allowed us to (i) gather a broader range of perspectives than previous studies and (ii) explore differences between groups. Given participants are more likely to feel comfortable sharing their perspectives in a homogenous group [32], the stratification also maximised the chances that the discussions held were rich and participant responses were genuine.

Conclusion

Results from the present study exploring perspectives on Australia's e-cigarette regulations and recommendations for further regulations suggest that many believe the current prescription model for nicotine e-cigarette products to be effective when enforced appropriately. Several concerns were raised about the availability of non-nicotine e-cigarettes, suggesting that planned reforms to restrict the supply of these products are likely to be well-received. Given many of the recommendations made by participants for effective regulations were those announced by the Federal Government in the months after this study was conducted, the proposed reforms are likely to be met with little objection from the public. However, ensuring the reforms are complemented by efforts to support those who are addicted to e-cigarettes or who are using e-cigarettes to cope with mental health struggles is critical.

Ethical approval: The University of Melbourne Human Research Ethics Committee (2023-24865-38247-10).

Funding statement: This work was supported by a National Health and Medical Research Council Investigator Grant (APP1194713). The funding source had no involvement in study design; in the collection, analysis and interpretation of data; in the writing of the article; and in the decision to submit the article for publication.

Declarations of interest: None.

Data availability: Upon reasonable request. Data will not be shared with those affiliated with the tobacco or e-cigarette industries.

For peer review only

For peer review only - http://bmjopen.bmj.com/site/about/guidelines.xhtml

Author contributions: M.I.J: Funding acquisition, conceptualization, methodology, writing – original manuscript, review and editing, supervision, project administration. S.J.L.Y: Methodology, writing – reviewing and editing. M.E.B: Data curation, formal analysis, writing – original manuscript, reviewing, editing.

For peer review only

For peer review only - http://bmjopen.bmj.com/site/about/guidelines.xhtml

References

1. Filippidis, F.T., Laverty, A.A., Gerovasili, V., and Vardavas, C.I., Two-year trends and predictors of e-cigarette use in 27 European Union member states. *Tobacco Control*, 2017; 26(1): p. 98-104.
2. Obisesan, O.H., Osei, A.D., Uddin, S.M.I., et al., Trends in e-cigarette use in adults in the United States, 2016-2018. *JAMA Internal Medicine*, 2020; 180(10): p. 1394-1398.
3. Greenhalgh, E., Bain, E., Jenkins, S., and Scollo, M., *Prevalence of e-cigarette use.*, in *Tobacco in Australia: Facts and issues*, E. Greenhalgh, M. Scollo, and M. Winstanley, Editors. 2023, Cancer Council Victoria: Melbourne.
4. Wakefield, M., Haynes, A., Tabbakh, T., et al., *Current vaping and current smoking in the Australian population ages 14+ years: February 2018-March 2023*. 2023: Centre for Beahvioural Research in Cancer, Cancer Council Victoria: Melbourne, Australia.
5. Berry, K.M., Fetterman, J.L., Benjamin, E.J., et al., Association of electronic cigarette use with subsequent initiation of tobacco cigarettes in US youths. *JAMA Network Open*, 2019; 2(2): p. e187794-e187794.
6. Banks, E., Yazidjoglou, A., Brown, S., et al., Electronic cigarettes and health outcomes: Umbrella and systematic review of the global evidence. *Medical Journal of Australia*, 2023; 218(6): p. 267-275.
7. Lechner, W.V., Janssen, T., Kahler, C.W., et al., Bi-directional associations of electronic and combustible cigarette use onset patterns with depressive symptoms in adolescents. *Preventive Medicine*, 2017; 96: p. 73-78.
8. World Health Organization, Backgrounder on WHO report on regulation of e-cigarettes and similar products. *Geneva: Noncommunicable Diseases And Mental Health*, 2014.
9. Jongenelis, M.I., Kameron, C., Rudaizky, D., and Pettigrew, S., Support for e-cigarette regulations among Australian young adults. *BMC Public Health*, 2019; 19(1): p. 67.
10. Greenhalgh, E.M., Smith, L., Grace, C., and Scollo, M., eds. *18.13 E-cigarettes and other alternative nicotine products: Legal status in Australia*. Tobacco in Australia: Facts and issues, ed. E.M. Greenhalgh, M.M. Scollo, and M.H. Winstanley. 2022, Cancer Council Victoria: Melbourne.

For peer review only - http://bmjopen.bmj.com/site/about/guidelines.xhtml

11. Dessaix, A., Jardine, E., Freeman, B., and Kameron, C., Undermining Australian controls on electronic nicotine delivery systems: Illicit imports and illegal sales. *Tobacco Control*, 2022; 31(6): p. 689-690.

12. Department of Health and Aged Care. About vaping and e-cigarettes. 2023; Available from: https://www.health.gov.au/topics/smoking-vaping-and-tobacco/about-vaping.

13. Therapeutic Goods Administration. New regulation of vapes starting January 2024. 2023; Available from: https://www.tga.gov.au/news/media-releases/new-regulation-vapes-starting-january-2024.

14. Diepeveen, S., Ling, T., Suhrcke, M., et al., Public acceptability of government intervention to change health-related behaviours: A systematic review and narrative synthesis. *BMC Public Health*, 2013; 13(1): p. 756.

15. Proctor, E., Silmere, H., Raghavan, R., et al., Outcomes for implementation research: conceptual distinctions, measurement challenges, and research agenda. *Administration and Policy in Mental Health and Mental Health Services Research*, 2011; 38(2): p. 65-76.

16. Bain, E., Mitsopoulos, E., and Durkin, S., *Perceptions of and support for policies concerning the availability and use of e-cigarettes among Australian adults, 2021 and 2022.* 2023; Centre for Behavioural Research in Cancer, Cancer Council Victoria: Melbourne, Australia.

17. Fraser, D., Weier, M., Keane, H., and Gartner, C., Vapers' perspectives on electronic cigarette regulation in Australia. *International Journal of Drug Policy*, 2015; 26(6): p. 589-594.

18. Sekhon, M., Cartwright, M., and Francis, J.J., Acceptability of healthcare interventions: An overview of reviews and development of a theoretical framework. *BMC Health Services Research*, 2017; 17(1): p. 1-13.

19. Smith, L.E., Amlôt, R., Lambert, H., et al., Factors associated with adherence to self-isolation and lockdown measures in the UK: A cross-sectional survey. *Public Health*, 2020; 187: p. 41-52.

20. Braun, V. and Clarke, V., Using thematic analysis in psychology. *Qualitative Research in Psychology*, 2006; 3(2): p. 77-101.

21. Braun, V. and Clarke, V., Reflecting on reflexive thematic analysis. *Qualitative Research in Sport, Exercise And Health*, 2019; 11(4): p. 589-597.

For peer review only - http://bmjopen.bmj.com/site/about/guidelines.xhtml

22. Smith, B. and McGannon, K.R., Developing rigor in qualitative research: Problems and opportunities within sport and exercise psychology. *International Review of Sport and Exercise Psychology*, 2018; 11(1): p. 101-121.

23. Selamoglu, M., Erbas, B., Kasiviswanathan, K., and Barton, C., General practitioners' knowledge, attitudes, beliefs and practices surrounding the prescription of e-cigarettes for smoking cessation: A mixed-methods systematic review. *BMC Public Health*, 2022; 22(1): p. 2415.

24. Stone, E. and Marshall, H., Electronic cigarettes in physician practice: A complex debate. *Internal Medicine Journal*, 2019; 49(4): p. 438-445.

25. Jongenelis, M.I., Robinson, A., Hughes, A., and Pettigrew, S., Perceptions of a prescription model for accessing nicotine vaping products: An examination of submissions made by self-reported e-cigarette users to an Australian consultation. *Health Promotion International*, 2023; 38(4): p. daad080.

26. Chivers, E., Janka, M., Franklin, P., et al., Nicotine and other potentially harmful compounds in "nicotine-free" e-cigarette liquids in Australia. *Med J Aust*, 2019; 210(3): p. 127-8.

27. Nguyen Zarndt, A., Donaldson, E.A., Bernat, J.K., et al., Adult use of and transitions from nicotine and non-nicotine-containing e-cigarettes: Data from the Population Assessment of Tobacco and Health (PATH) study, 2013-2016. *Nicotine and Tobacco Research*, 2020; 22(7): p. 1225-1229.

28. Cuccia, A.F., Patel, M., Amato, M.S., et al., Quitting e-cigarettes: Quit attempts and quit intentions among youth and young adults. *Preventive Medicine Reports*, 2021; 21: p. 101287.

29. Palmer, A.M., Price, S.N., Foster, M.G., et al., Urgent Need for novel investigations of treatments to quit e-cigarettes: Findings from a systematic review. *Cancer Prevention Research*, 2022; 15(9): p. 569-580.

30. Elo, S., Kääriäinen, M., Kanste, O., et al., Qualitative content analysis: A focus on trustworthiness. *SAGE Open*, 2014; 4(1): p. 2158244014522633.

31. Nowell, L.S., Norris, J.M., White, D.E., and Moules, N.J., Thematic analysis: Striving to meet the trustworthiness criteria. *International Journal of Qualitative Methods*, 2017; 16(1): p. 1609406917733847.

32. Krueger, R.A., Casey, M.A. *Focus groups: A practical guide for applied research*. 2014: Thousand Oaks, CA: Sage Publications.

For peer review only - http://bmjopen.bmj.com/site/about/guidelines.xhtml

For peer review only

For peer review only - http://bmjopen.bmj.com/site/about/guidelines.xhtml

Supplementary Material

Table S1. Coding Hierarchy

Parent code	Child code #1	Child code #2	Child code #3
Perspectives on current regulations	Perspectives on the prescription model	Positive sentiment	Should reduce use among young people and those who have never smoked
		Negative sentiment	E-cigarettes are not a viable quitting aid
			Barriers to access vs. the system is easy to manipulate
			Illusion of product safety
	Perspectives on the availability of non-nicotine e-cigarette products	Positive sentiment	Libertarian views – adults should be allowed to choose what they consume
			Non-nicotine products as potential quitting aids
		Negative sentiment	Safety and addiction concerns
			Nicotine content concerns
			Gateway to nicotine use
			Difficult to enforce differential regulations for nicotine and non-nicotine products
Recommendations for potential regulations	Demand factors	Product and packaging	Health warnings on product packaging

For peer review only

			Plain packaging
			Remove flavours
			Provide detailed information about the contents of e-cigarettes
		Increase price of e-cigarettes	-
		Increase the number of vape-free areas	-
		Limit advertising	Reduce visibility
			Stop appealing to children/young people
	Supply factors	Ban e-cigarettes	Ban importation of all e-cigarettes
			Ban disposable e-cigarettes
		Make e-cigarettes less available to children	-
		Legalise access to e-cigarettes for adults	-
	Miscellaneous	Increase support for mental health and addiction	-
		Enforce regulations	-

For peer review only

Standards for Reporting Qualitative Research (SRQR)*

http://www.equator-network.org/reporting-guidelines/srqr/

Title and abstract

	Page/line no(s).
**Title** - Concise description of the nature and topic of the study Identifying the study as qualitative or indicating the approach (e.g., ethnography, grounded theory) or data collection methods (e.g., interview, focus group) is recommended	Pages 1 and 2
**Abstract** - Summary of key elements of the study using the abstract format of the intended publication; typically includes background, purpose, methods, results, and conclusions	Page 2

Introduction

**Problem formulation** - Description and significance of the problem/phenomenon studied; review of relevant theory and empirical work; problem statement	Page 4 and 5
**Purpose or research question** - Purpose of the study and specific objectives or questions	Page 5

Methods

**Qualitative approach and research paradigm** - Qualitative approach (e.g., ethnography, grounded theory, case study, phenomenology, narrative research) and guiding theory if appropriate; identifying the research paradigm (e.g., postpositivist, constructivist/ interpretivist) is also recommended; rationale**	Page 7
**Researcher characteristics and reflexivity** - Researchers' characteristics that may influence the research, including personal attributes, qualifications/experience, relationship with participants, assumptions, and/or presuppositions; potential or actual interaction between researchers' characteristics and the research questions, approach, methods, results, and/or transferability	Pages 6 and 7
**Context** - Setting/site and salient contextual factors; rationale**	Pages 5-7
**Sampling strategy** - How and why research participants, documents, or events were selected; criteria for deciding when no further sampling was necessary (e.g., sampling saturation); rationale**	Page 6
**Ethical issues pertaining to human subjects** - Documentation of approval by an appropriate ethics review board and participant consent, or explanation for lack thereof; other confidentiality and data security issues	Page 7
**Data collection methods** - Types of data collected; details of data collection procedures including (as appropriate) start and stop dates of data collection and analysis, iterative process, triangulation of sources/methods, and modification of procedures in response to evolving study findings; rationale**	Pages 6 and 7

For peer review only - http://bmjopen.bmj.com/site/about/guidelines.xhtml

**Data collection instruments and technologies** - Description of instruments (e.g., interview guides, questionnaires) and devices (e.g., audio recorders) used for data collection; if/how the instrument(s) changed over the course of the study	Pages 6 and 7
**Units of study** - Number and relevant characteristics of participants, documents, or events included in the study; level of participation (could be reported in results)	Pages 6 and 7
**Data processing** - Methods for processing data prior to and during analysis, including transcription, data entry, data management and security, verification of data integrity, data coding, and anonymization/de-identification of excerpts	Page 7
**Data analysis** - Process by which inferences, themes, etc., were identified and developed, including the researchers involved in data analysis; usually references a specific paradigm or approach; rationale**	Page 7
**Techniques to enhance trustworthiness** - Techniques to enhance trustworthiness and credibility of data analysis (e.g., member checking, audit trail, triangulation); rationale**	Page 7

Results/findings

**Synthesis and interpretation** - Main findings (e.g., interpretations, inferences, and themes); might include development of a theory or model, or integration with prior research or theory	Pages 8-12
**Links to empirical data** - Evidence (e.g., quotes, field notes, text excerpts, photographs) to substantiate analytic findings	Page 8-12

Discussion

**Integration with prior work, implications, transferability, and contribution(s) to the field -** Short summary of main findings; explanation of how findings and conclusions connect to, support, elaborate on, or challenge conclusions of earlier scholarship; discussion of scope of application/generalizability; identification of unique contribution(s) to scholarship in a discipline or field	Pages 12-16
**Limitations** - Trustworthiness and limitations of findings	Page 15

Other

**Conflicts of interest** - Potential sources of influence or perceived influence on study conduct and conclusions; how these were managed	Page 16
**Funding** - Sources of funding and other support; role of funders in data collection, interpretation, and reporting	Page 16

*The authors created the SRQR by searching the literature to identify guidelines, reporting standards, and critical appraisal criteria for qualitative research; reviewing the reference lists of retrieved sources; and contacting experts to gain feedback. The SRQR aims to improve the transparency of all aspects of qualitative research by providing clear standards for reporting qualitative research.

For peer review only

For peer review only - http://bmjopen.bmj.com/site/about/guidelines.xhtml

**The rationale should briefly discuss the justification for choosing that theory, approach, method, or technique rather than other options available, the assumptions and limitations implicit in those choices, and how those choices influence study conclusions and transferability. As appropriate, the rationale for several items might be discussed together.

Reference:

O'Brien BC, Harris IB, Beckman TJ, Reed DA, Cook DA. **Standards for reporting qualitative research: a synthesis of recommendations.** *Academic Medicine*, Vol. 89, No. 9 / Sept 2014
DOI: 10.1097/ACM.0000000000000388

For peer review only
